# New frontiers in the structural analysis of nanomaterials: a deep-learning leap to bridge the gap in crystalline order

**DOI:** 10.1107/S2052252526008444

**Published:** 2026-10-08

**Authors:** Fabrizio Mastrolorito, Mauro De Feudis, Fulvio Ciriaco, Rocco Caliandro, Orazio Nicolotti, Angela Altomare

**Affiliations:** a Department of Pharmacy – Pharmaceutical Sciences University of Bari Aldo Moro Bari Italy; b Institute of Crystallography, CNR, Bari, Italy; c Department of Chemistry University of Bari Aldo Moro Italy; Tsinghua University, China

**Keywords:** structural characterization, nanomaterials, powder diffraction, peak resolution, neural networks, computational modeling, materials modeling, nanostructure, crystalline order, deep learning

## Abstract

High-resolution crystal structure determination using X-ray, electron or neutron diffraction is often limited by poor crystallinity, small crystallite size and instrumental factors, which cause significant diffraction peak broadening and a consequent loss of information. This challenge is particularly severe for nanocrystalline materials, whose X-ray powder diffraction (XRPD) profiles exhibit extensive peak overlap, thereby hindering the reliable extraction of structural information through conventional crystallographic methods. We present here a novel deep-learning-based approach to increase the probability of determining the structure of nanocrystals. A deep-learning model is trained to transform simulated diffraction patterns, degraded by reduced crystalline order, into optimal profiles representative of ideal perfectly crystalline materials. Instead of attempting direct structure inference, this approach improves data by making them better suited for standard crystallographic analyses. The resulting pipeline is both robust and versatile, demonstrating consistent performance across varying crystallite sizes on simulated profiles. The procedure has also been successfully applied to experimental data. To our knowledge, this is the first application of deep learning to enhance experimental XRPD data, representing a breakthrough in the structural characterization of nanocrystalline materials, specifically by enabling progress in difficult cases where atomic-level structure determination is hindered by poor diffraction data.

## Abstract 2

We address the challenge of solving *ab initio* crystal structures from X-ray powder diffraction (XRPD) data acquired from nanomaterials. Our approach employs artificial intelligence to pre-process XRPD profiles with the aim of recovering information lost by lack of crystalline order.

## 1. Introduction

Atomic resolution insights into matter can be obtained through the diffraction of X-rays, electrons or neutrons. Optimal diffraction requires long-range periodicity where atoms of the compound under study are arranged according to a well defined space-group symmetry and repeat themselves throughout the crystal lattice via three-dimensional translation of the unit cell. Such ordered structures are produced by crystallization processes, and are governed by thermodynamics, kinetics, and, in many cases, hydro­dynamic conditions. However, the outcomes of these processes are not fully under control, given the large number of variables involved.

The advent of nanotechnology has revealed that nano­materials, and specifically nanocrystalline materials (*i.e.* materials with crystallite size of only a few nanometres), exhibit relevant properties that differ from those of their bulk counterparts. Thus, controlling and limiting crystal size can be advantageous for applications. Nevertheless, nanocrystalline materials still need to be characterized structurally at atomic resolution to engineer their structure–function relationships. Unfortunately, diffraction from nanometre-sized crystallites produces broad and overlapping Bragg peaks, making difficult the analysis of diffraction patterns with standard crystallographic methods and hindering the extraction of detailed structural information.

However, peak broadening in powder diffraction is a key source of microstructural information, increasingly exploited to characterize crystallite size and morphology, microstrain, and defect populations (Lutterotti & Scardi, 1990 ▸; Scardi *et al.*, 2018 ▸). The various microstructural contributions affect diffraction profiles in different ways. A distinction is commonly made between isotropic broadening, for which the peak width varies smoothly as a function of the diffraction angle 2ϑ, and anisotropic broadening, typically associated with anisotropic crystallite shape or characteristic fluctuations in peak widths among neighboring reflections in reciprocal space.

Furthermore, in materials with only short-range order, such as highly disordered, paracrystalline, or amorphous systems, long-range periodicity is lost, leading to diffuse scattering instead of sharp Bragg reflections.

At the same time, in the case of crystals with long-range order, instrumental factors can limit the quality of recorded diffraction data, due to imperfections in the optics of the incident or diffracted beams, or in the detection system adopted to measure the patterns. These effects produce a decrease in the peak resolution, which in turn results in a broadening of the Bragg peaks.

In particular, instrumental broadening arises from the finite resolution of the diffractometer and is affected by factors such as source size, wavelength distribution, beam divergence, slit geometry, monochromators, detector characteristics, and sample geometry. The angular dependence of the instrumental resolution function is commonly described using the Caglioti relation (Caglioti *et al.*, 1958 ▸) or, more rigorously, through fundamental-parameter approaches that model each instrumental contribution explicitly.

In summary, both sample-dependent and experimental effects often limit the possibility of having access to a high-resolution view of atomic order.

However, the crystal structure cannot be directly determined from a diffraction experiment. Instead, it is inferred through a computational process known as ‘phasing’, in which a phase value is assigned to each measured diffracted intensity (*i.e.* reflection). Phasing processes are developed in the framework of crystallographic methods, which investigate how to obtain structural information from a diffraction pattern. They proceed through the following steps:

(i) Indexing: assigning the Miller indices to each reflection by determining the crystal unit cell.

(ii) Extracting intensities: deriving diffraction intensities of each reflection to determine amplitudes.

(iii) Phasing: assigning and refining phase values.

(iv) Model building and refinement: determining the structural model and refining its parameters.

(v) Validating: assessing the quality and reliability of the final structural model.

These steps are routinely carried out by computer programs specifically developed for the automatic crystal structure determination. Their success strongly depends on the type and the quality of the collected diffraction data, which are directly related to the properties of the crystalline sample.

Determining crystal structures from X-ray powder diffraction (XRPD) data is much more challenging than from single-crystal data. Peak overlap, especially in the presence of significant peak broadening, complicates the assignment of diffraction intensities to individual reflections. Both limited crystalline order and experimental aberrations reduce peak resolution in XRPD profiles, negatively affecting all subsequent steps of structure determination, from indexing to refinement. For nanomaterials, substantial peak broadening and the resulting severe peak overlap makes structural characterization by standard crystallographic software extremely difficult. Performances of current crystallographic software for structural solution from XPRD data, such as *EXPO* (Altomare *et al.*, 2013 ▸), *TOPAS* (Coelho, 2018 ▸), *DASH* (David *et al.*, 2006 ▸) and *SUPERFLIP* (Palatinus & Chapuis, 2007 ▸), are strongly limited in the case of crystallites of nanometre size and low peak resolution data, which prevent reliable structural characterization for such materials.

Several pre-processing approaches have been proposed to improve the quality of XRPD profiles, such as correcting instrumental aberrations by deconvolution (Ida & Toraya, 2002 ▸), or improving the diffraction signal by filtering (Ladisa *et al.*, 2007 ▸). However, none of these has produced a major breakthrough.

A transformative opportunity lies in machine learning, which has recently been making improvements in various fields of crystallographic analysis (Agrawal & Choudhary, 2016 ▸; Liu *et al.*, 2020 ▸; Long *et al.*, 2022 ▸; Su *et al.*, 2024 ▸). In particular, convolutional neural networks can resolve overlapping XRPD peaks (Liu *et al.*, 2022 ▸; Xie & Grossman, 2018 ▸) and attention mechanisms have shown strong capabilities in performing classification tasks applied to multi-phase polycrystalline samples (Cao *et al.*, 2025 ▸; Chen *et al.*, 2024 ▸; Choudhary *et al.*, 2022 ▸; Dong *et al.*, 2021 ▸; Lee *et al.*, 2020 ▸; Maffettone *et al.*, 2021 ▸; Salgado *et al.*, 2023 ▸; Schleder *et al.*, 2019 ▸; Szymanski *et al.*, 2023 ▸; Wang *et al.*, 2020 ▸; Zhang *et al.*, 2024 ▸; Zheng *et al.*, 2023 ▸).

In the field of crystal structure solution, deep learning has revolutionized the structure determination process of small molecules, being able to assign a phase value to low-resolution reflections (Carrozzini *et al.*, 2025*a* ▸,*b* ▸; Larsen *et al.*, 2024 ▸); to automatically deconvolute a Patterson map (Pan *et al.*, 2023 ▸); or to directly infer the crystal structure from XRPD profiles (Guo *et al.*, 2025 ▸; Lee *et al.*, 2022 ▸; Park *et al.*, 2017 ▸; Vecsei *et al.*, 2019 ▸) or by combining X-ray and electron diffraction measurements (Aguiar *et al.*, 2019 ▸).

Building on this progress, we have devised a new procedure based on deep learning for pre-processing XRPD profiles. The method aims to bridge the gap caused by sample limitations, transforming a real diffraction profile with broad peaks into one with narrower peaks that represents an ideal crystalline material.

This marks a significant advance in applying machine learning to structural analysis. For the first time, deep learning is trained to compensate for reduced crystalline order, allowing structure solution by standard crystallographic methods. Rather than directly associating a structural model with the measured XRPD profile, our approach focuses on enhancing the information content of the data by improving peak resolution, thereby making the dataset better suited for traditional structure determination.

This approach provides a practical balance: it avoids the significant challenges of directly determining the crystal structure by automatically decoding a low-quality experimental diffraction profile, while advancing beyond existing pre-processing methods based solely on statistical analysis.

Our pipeline, where the XRPD profile is first pre-processed by a deep-learning model and then analyzed by crystallographic software for the crystal structure determination, is both flexible and robust. The proposed approach spans different levels of peak resolution, corresponding to materials with varying crystallite sizes, and shows consistent performances on simulated profiles. Additionally, it has also been successfully applied to experimental data.

## 2. Methods

### 2.1. Training data

#### 2.1.1. Crystal structure selection

Our deep-learning architecture was trained on simulated powder diffraction patterns representative of real cases. To generate the patterns, we selected a large set of structures from the Crystallography Open Database (COD) (Downs & Hall-Wallace, 2003 ▸; Gražulis *et al.*, 2009 ▸).

Determining crystal structures from XRPD data of organic compounds remains challenging, even for highly crystalline samples with moderate crystallite size. This difficulty is due to several interrelated factors: these compounds frequently crystallize in low-symmetry space groups with large unit cells, resulting in closely spaced reflections and peak overlap; they are mainly composed of light atoms with weak X-ray scattering power, producing low diffraction intensities and limited experimental data resolution; and they often exhibit preferred orientation, particularly in needle- or plate-like crystals, introducing systematic intensity errors. Additional complications arise from flexible molecular structures, the presence of solvent molecules, and structural disorder. Furthermore, each step in structure solution presents significant challenges due to the inherent limitations of powder diffraction. Therefore, to increase the complexity of our study, we selected organic compounds, with volumes up to 2000 Å^3^ containing one or more of the following atomic elements: C, H, N and O.

Structures were imported using a tool in the *EXPO* software capable of directly retrieving CIFs from COD. In total, 33 125 structures met these criteria and were used to generate our training set. For each crystal structure, we simulated ten corresponding powder diffraction profiles, each corresponding to a different crystallite size ranging from small to large. The smallest size produces a pattern suitable for standard XRPD structure-solution programs, while the largest simulates conditions where severe peak broadening renders analysis inadequate for structural studies, from the indexing step to the model building and refinement. Importantly, our deep-learning model was trained on data derived from structures that are, on average, challenging to solve even when crystallite size is not a limiting factor.

#### 2.1.2. Generation of simulated diffraction profiles

The training data for our neural network consist of simulated XRPD patterns generated from CIFs of sample crystal structures selected from COD. A tool implemented in *EXPO* was used to generate the patterns, allowing peak shapes to be modeled with the Pearson VII function and enabling adjustment of parameters such as the radiation wavelength, pattern range, step width, and the full width at half-maximum (FWHM). To generate XRPD data comparable to realistic experimental patterns (Lee *et al.*, 2023 ▸), both the peak shapes and the statistical uncertainties of the data were modeled. The FWHM was modeled using the Caglioti formula as a function of ϑ (Caglioti *et al.*, 1958 ▸), while the parameter of the Pearson VII function was kept constant with respect to ϑ but allowed to vary between different structures, taking values of 1, 2 or 11. In addition, statistical noise was introduced into the calculated intensities following a Poisson distribution, which was approximated by a Gaussian distribution as the intensity increased (Mendenhall, 2018 ▸). For all generated patterns, Cu *K*α_1_ radiation (λ = 1.54056 Å) was assumed, the step width was set to 0.02° (2ϑ), and the range was defined between 5° and 80° (2ϑ), corresponding to an experimental data resolution of 1.2 Å. This value is lower than the ideal 1.0 Å atomic resolution and more realistically approximates experimental data typically collected from powder samples, particularly from organic compounds.

For each CIF, ten diffraction profiles (numbered from 1 to 10) were generated by varying the FWHM parameters, so that pattern 1 exhibited a small FWHM value of ∼0.1° in 2ϑ at low ϑ, gradually increasing up to the pattern 10, which displayed an FWHM value of ∼1.0° (2ϑ) at low ϑ. The dependence of the FWHM on ϑ was simulated by varying the three profile parameters *W*, *V* and *U* for each generated diffraction pattern. Specifically, random values of *W*, *V* and *U* were assigned within the ranges reported in Table 1 ▸. Distinct parameter ranges were defined for each of the ten diffraction profiles (Profiles 1–10) generated from a given CIF, resulting in different peak-broadening characteristics across the simulated patterns.

The choice of FWHM values follows the rationale of the Debye–Scherrer equation (Scherrer, 1918 ▸), which relates the peak width in a powder diffraction profile to the mean size of the crystalline domains of the sample:
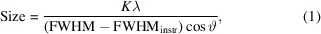
where *K* is a dimensionless shape factor (typically ∼0.9, but varies with crystallite morphology), 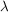 is the radiation wavelength of the primary beam, FWHM is the peak width (in radians) and FWHM_instr_ accounts for instrumental broadening, (*i.e.* refers to the FWHM of peaks of a calibration highly crystalline compound). Equation (1) thus combines both the peak broadening due to finite crystal size (Size) and the contributions from the experimental setup, such as focusing optics and detector resolution (FWHM_instr_), to describe the observed peak width in a powder diffraction profile.

In this study, the Debye–Scherrer equation was used to select appropriate FWHM values for generating simulated XRPD profiles, in order to efficiently sample a realistic range of crystallite sizes commonly found in synthesized (nano)materials. Assuming negligible instrumental broadening (FWHM_instr_ ≃ 0), the selected FWHM values correspond to crystallite domain sizes ranging from ∼10 to 100 nm.

The final database consists of 331 250 XRPD patterns, which were used to feed our deep-learning model.

**Figure 1:**
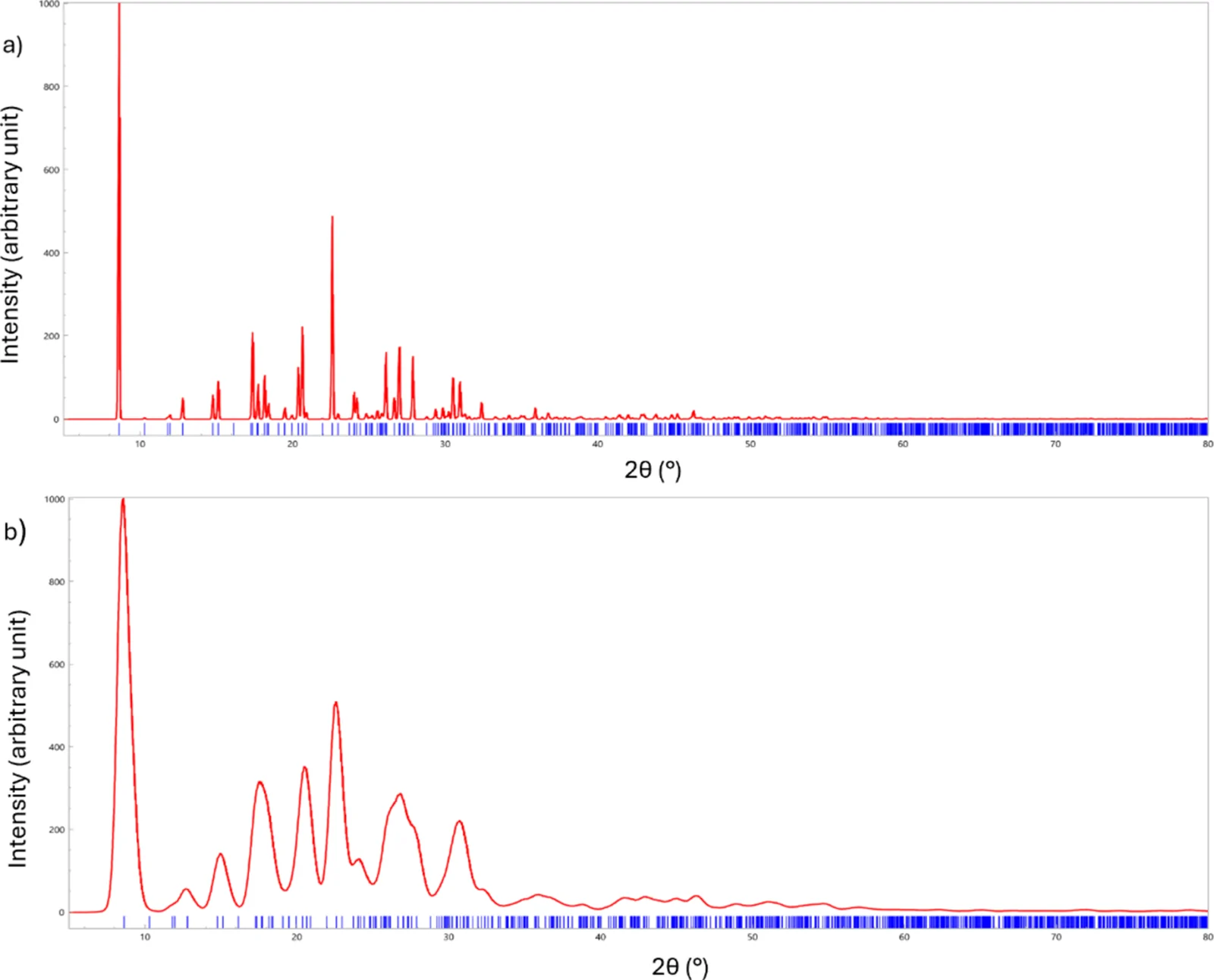
Simulated X-ray diffraction profiles of C_18_H_14_N_4_O_4_ (Lee *et al.*, 2011 ▸), calculated by using a full width half-maximum parameter (FWHM) of (*a*) ∼0.1° and (*b*) ∼1.0°. The small blue vertical bars correspond to positions of reflections.

As an example, Fig. 1 ▸ shows two diffraction profiles from our training set, both generated from the same crystal structure (C_18_H_14_N_4_O_4_) (Lee *et al.*, 2011 ▸). They correspond to (*a*) pattern 1, with an FWHM of ∼0.1° (2ϑ), and (*b*) pattern 10, with an FWHM of ∼1.0° (2ϑ), corresponding to peak widths of ∼5 steps in 2ϑ and 50 steps in 2ϑ, respectively. These values roughly correspond to crystallite sizes of 100 nm and 10 nm, respectively. The small blue vertical bars at the bottom of each profile indicate the positions of reflections of the sample crystal structure. The figure clearly illustrates the increasing degree of peak overlap with broader peak widths. For experimental patterns with a large FWHM, as shown in Fig. 1 ▸(*b*), the low accuracy of diffraction peak positions and intensities hinders the successful completion of each step in the structure-solution process.

In addition, the potential presence of peak asymmetry was also considered. To this aim, a contribution was added to the profile function using the asymmetry function proposed by Bérar & Baldinozzi (1993 ▸), with four parameters A0, A1, B0 and B1, which were assigned empirically. Specifically, A0 was randomly varied within the range of −0.15–0.15, B0 was set equal to 0.2 times A0, and both A1 and B1 were fixed at zero.

### 2.2. Experimental data

#### 2.2.1. Crystal structure selection

Our deep-learning model was further validated by using 33 experimental powder diffraction profiles retrieved from COD. These profiles were not included in the training set. They correspond to crystal structures whose main crystal-chemical information is reported in Table S1 of the supporting information, along with their COD codes. All structures satisfy the same criteria adopted for the generation of the simulated data: organic compounds with unit-cell volumes up to 2000 Å^3^ and containing one or more of the elements C, H, N and O. These were the only structures in COD verifying these criteria and for which experimental powder diffraction patterns were available. From the original publications reporting these structures, we found that they had been solved using different software packages and structure-solution strategies.

#### 2.2.2. Pre-processing

Experimental profiles need to be pre-processed prior to input into our deep-learning model in order to reproduce the features of the profiles used during training. Pre-processing steps include background subtraction, a fixed 2ϑ step of 0.02° over the angular range 5°–80°, and use of Cu *K*α_1_ radiation. All pre-processing steps were performed using the *RootProf* computer program (Caliandro & Belviso, 2014 ▸; Mazzone *et al.*, 2023 ▸). Background subtraction was carried out using the sensitive nonlinear iterative peak (SNIP) clipping algorithm (Ryan *et al.*, 1988 ▸) with a window size of 10. The fixed step was obtained by interpolating the profiles through a cubic spline function. Profiles collected with X-ray wavelengths different from Cu *K*α_1_ were converted to the corresponding 2ϑ positions for Cu *K*α_1_ radiation using Bragg’s law.

### 2.3. Deep-learning architecture

**Figure 2:**
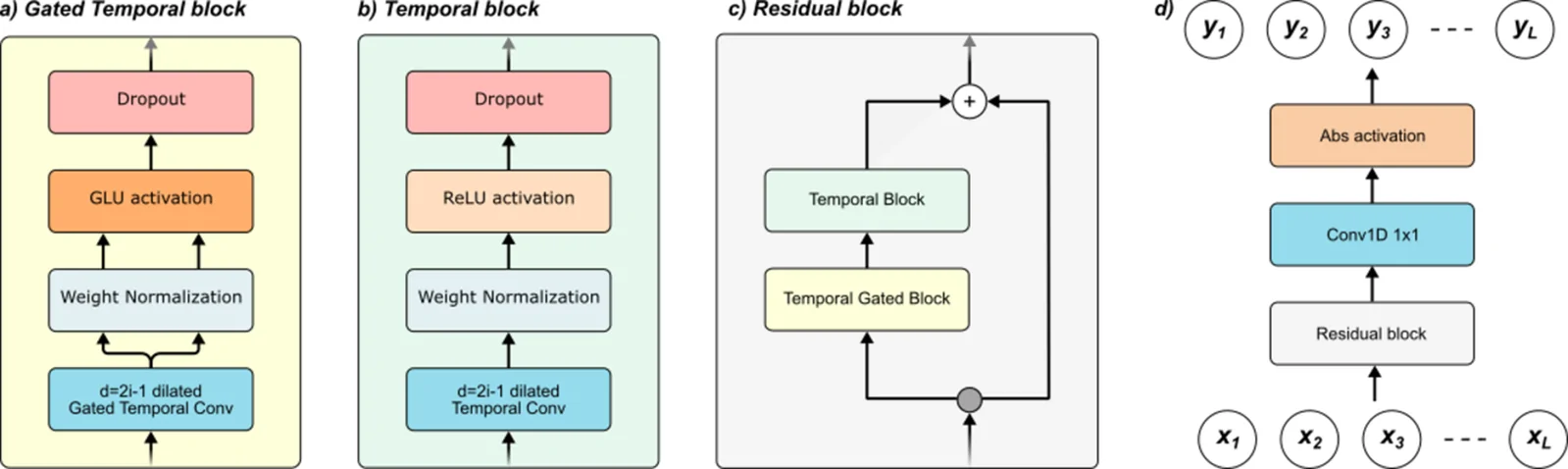
Architecture of the TCN used in this study. The model consists of (*a*) a gated temporal block and (*b*) a temporal block, both composed of dilated convolutional layers and stacked to form (*c*) a residual block where a residual connection is also involved. (*d*) The input array (*X*) is processed through residual blocks, followed by a Conv1D block and absolute-value activation function, producing the output array (*Y*).

To model the dependencies within the profile diffraction data, a temporal convolutional network (TCN) was adopted. The implementation is based on acknowledged TCN architecture (Bai *et al.*, 2018 ▸), whose residual blocks are stacked on each other for each series (Fig. 2 ▸).

In the present study, each residual block consists of a temporal gated block where a gated linear unit (GLU) (Dauphin *et al.*, 2017 ▸) is employed as an activation function and then a temporal block where the activation function is a rectified linear unit (ReLU). The final output was obtained by applying the absolute value function to avoid failure outputs due to our custom loss function implementation (Tan & Lim, 2019 ▸).

All layers were initialized using the Xavier uniform method (Glorot & Bengio, 2010 ▸) and the resulting weights were adopted to normalize input data values for each convolutional layer.

This architecture, referred to as *POWnet*, is designed to enhance peak resolution by processing, for each crystal structure, a sequence of diffraction profiles with varying FWHM values and therefore differing peak resolutions. In this respect, although XRD patterns are not temporal signals, they can be treated as ordered one-dimensional sequences. Therefore, a TCN was adopted as a feature extractor irrespective of the temporal interpretation for which it was originally proposed. The use of dilated convolutions allows a better detection of both local peak characteristics and long-range correlations. Since an unclear relationship exists along the 2ϑ axis, non-causal convolutions were employed. Each diffraction point was thus allowed to exploit information from both neighboring directions, this being more suitable to explore the physical nature of XRD patterns. In this framework, the model was trained using profiles numbered from 2 to 10, with the highest-quality profile, numbered as 1, serving as the reference. Therefore, the dataset for deep-learning purpose was composed of 298 125 (33 125 × 9) data rows.

The initial dataset was partitioned into training (80%), validation (10%), and test (10%) sets to ensure a rigorous assessment of model performance and generalization to unseen data. All the profiles derived from a given structure are collected in the same split.

Model parameters were optimized on the training set by minimizing a sole chosen figure of merit among those defined in the next section (*R*_p_, *R*_wp_, Corr or Sim) during the learning process of 50 epochs. Different combinations of hyperparameters, including the available merit functions, were manually varied to explore a grid of values for model training.

The optimal model was selected based on the validation loss curve. The model was trained with num_channel = 3, dim_channel = 512, kernel_size = 9, batch_size = 256, dropout = 0.05 and lr = 0.001, by adopting the sole *R*_wp_ metric for optimization and training.

Model development and evaluation were performed in Python v3.13, with *PyTorch* v2.7 providing the tensor computation backend and *PyTorch Lightning* v2.5 enabling structured experiment management and distributed scaling. Model training was performed using eight GPUs in parallel to accelerate convergence and ensure computational efficiency. The implementation of the model architecture was based on the open-source code provided in the GitHub repository https://github.com/paul-krug/pytorch-tcn.

A random seed was drawn for each run to ensure reproducibility of the experiments, including dataset splitting, training and prediction.

### 2.4. Figures of merit

The following figures of merit were first applied for model training and then on the test set to evaluate the similarity between the *POWnet*-generated (or calculated, denoted by ‘*c*’) and the target (or observed, denoted by ‘*o*’) powder diffraction profiles:

(1) Crystallographic agreement factor, defined as
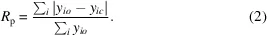


(2) Crystallographic weighted agreement factor, defined as
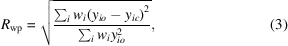
where the weight depends on the measurement uncertainty, 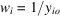, and the summation is over the number of profile intensities.

(3) Pearson correlation coefficient:
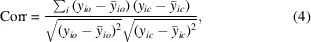
where 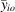 and 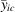 are the average values of the observed and calculated diffraction profiles, respectively.

(4) Weighted cross-correlation function:
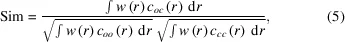
where 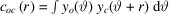 is the cross-correlation function between the powder diffraction profiles observed and calculated, while 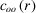 and 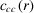 are their auto-correlation functions, defined analogously; *r* is the distance between two points in the powder diffraction profile and the weight function is a triangle function with half width *l*:
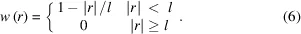
This function was originally developed by de Gelder *et al.* (2001 ▸) to compare powder diffraction profiles, and has the advantage of allowing limited peak misalignment thanks to the window *l* of its weight function, which can be adjusted to allow looser or more decisive similarity measures. We have set *l* = 0.1, since the angular step of our simulated diffraction profiles is 0.02°.

In the framework of our deep-learning model, each figure of merit was individually explored as a loss function to identify the most effective optimization function. Specifically, separate training experiments were performed using Corr, Sim, *R*_p_ and *R*_wp_ as the sole loss function. The results showed that Corr and Sim were not suitable optimization objectives, as they converged quite early while still yielding relatively high values for the *R*-based metrics. Among the tested figures of merit, *R*_wp_ consistently provided the best overall performance and led to the most accurate profile fitting. Therefore, *R*_wp_ was selected as the loss function to guide model optimization and training throughout this work.

### 2.5. Crystallographic tools for result evaluation

The goal of *POWnet* is transforming a diffraction pattern with overlapping broad peaks, which is typical of nanomaterials, into a pattern with sharper better-resolved peaks, as in the case of ideal and perfectly crystalline materials. The most effective way to test the performance of our deep-learning model is to submit the *POWnet* transformed profiles to each step of the structure-solution process and evaluate whether they can be correctly processed by crystallographic methods and software. The quality of the profiles supplied by deep learning was assessed through a multi-step procedure: (*a*) verifying the ability to index the profiles and determine the unit cell; (*b*) examining the accuracy of the extracted integrated diffraction intensities associated to each reflection; and (*c*) testing the capability to determine the three-dimensional crystal structure *ab initio*, that is, starting from only the experimental diffraction pattern, chemical formula, cell parameters and space group without any additional structural information. To this aim, the program *EXPO* was used with its standard protocols to perform the three processes for each transformed pattern provided by the network. The performance was evaluated according to the following criteria:

(*a*) The automatic *EXPO* indexing procedure typically generates a list of candidate unit cells, sorted by the figure of merit WRIP20, with the top ranked cell being the most likely correct solution (Altomare *et al.*, 2009 ▸). To verify if the correct cell is present in this list we adopted the following condition:
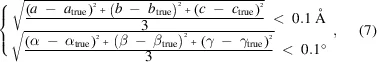
where *a*, *b*, *c*, α, β and γ are the unit-cell parameters of the candidate solution, and the subscript ‘true’ refers to the true cell parameters (*i.e.* those reported in the CIF). In equation (7) the cell lengths and angles are expressed in ångströms and degrees, respectively. Such an equation was applied after both the candidate solution cell and the true cell were transformed into their corresponding Niggli reduced cells (Niggli, 1928 ▸). The success of indexing was validated by monitoring the occurrence of a cell satisfying conditions (7), that is, a cell similar to the true one, within the top-ranked candidate solutions.

(*b*) The extraction through *EXPO* of integrated diffraction intensities, from which the experimental structure-factor moduli 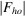 are derived, was assessed using the crystallographic agreement factor:
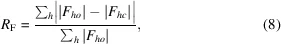
where 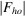 are the extracted amplitudes for each reflection *h*, and 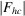 are the corresponding values calculated from the true structure reported in the CIF. Integrated intensities were extracted by *EXPO* using the Le Bail method (Le Bail *et al.*, 1988 ▸).

(*c*) The structural fragments located by the automatic *ab initio* structure-solution process in *EXPO* were evaluated by comparing the atomic positions in the *EXPO* models with the corresponding reference structures. The validation metric used was the CLP parameter, defined, for each test structure, as the ratio of correctly located atomic positions to the total number of atomic positions to be determined in the asymmetric unit, as specified in the CIF. An atomic position was considered correctly located if it laid within 0.6 Å of the true atomic position, taking into account symmetry-equivalent positions and allowed origin shifts.

### 2.6. Regression model

A regression model based on partial least squares as implemented in the *scikit-learn* Python library (Pedregosa *et al.*, 2011 ▸) was built to interpret the deep-neural-network results. Input data are the results obtained by the structure-solution procedure applied to the *POWnet* profiles, selected among those belonging to the test set. Each profile is described by a set of seven features (*X* variables), which characterize the corresponding crystal structure, and by two *Y* variables, which assess the quality of the structure-solution process. The number of components of the regression model has been optimized based on *R*^2^ and mean squared error (MSE) values calculated considering cross-validated predictions. The regression model was used to identify the key variables that control the structural solution process applied to the *POWnet* profiles. In addition, it was used to introduce a selection criterion to decide on which profiles to apply the *POWnet* pre-processing procedure, thus improving the efficiency of the proposed workflow.

## 3. Results

The input and output profiles of *POWnet* of the test set were evaluated in terms of their quality using the figures of merit described in Section 2.4. Subsequently the input and output profiles were assessed for their effectiveness in supporting the structure-solution process by considering key performance parameters associated with the indexing, extraction and solution steps listed in Section 2.5.

### 3.1. Simulated profiles

To evaluate the performance of the trained deep-learning model, a number of 3313 structures (that is, 10% of the total dataset) were randomly selected. Their corresponding simulated patterns calculated from the CIFs contained in COD were used for the analysis. Notably, this set was not included in the training set.

For each structure, the simulated profiles numbered 2 to 10 were used as input for *POWnet* to predict the corresponding high-resolution profile with an FWHM of ∼0.1° in 2ϑ. The model’s performance was evaluated using all figures of merit.

The performance values follow the expected trend, deteriorating as the resolution decreases (Table 2 ▸). However, the performance values remain above 0.92 when using Gelder’s metric as a reference.

**Figure 3:**
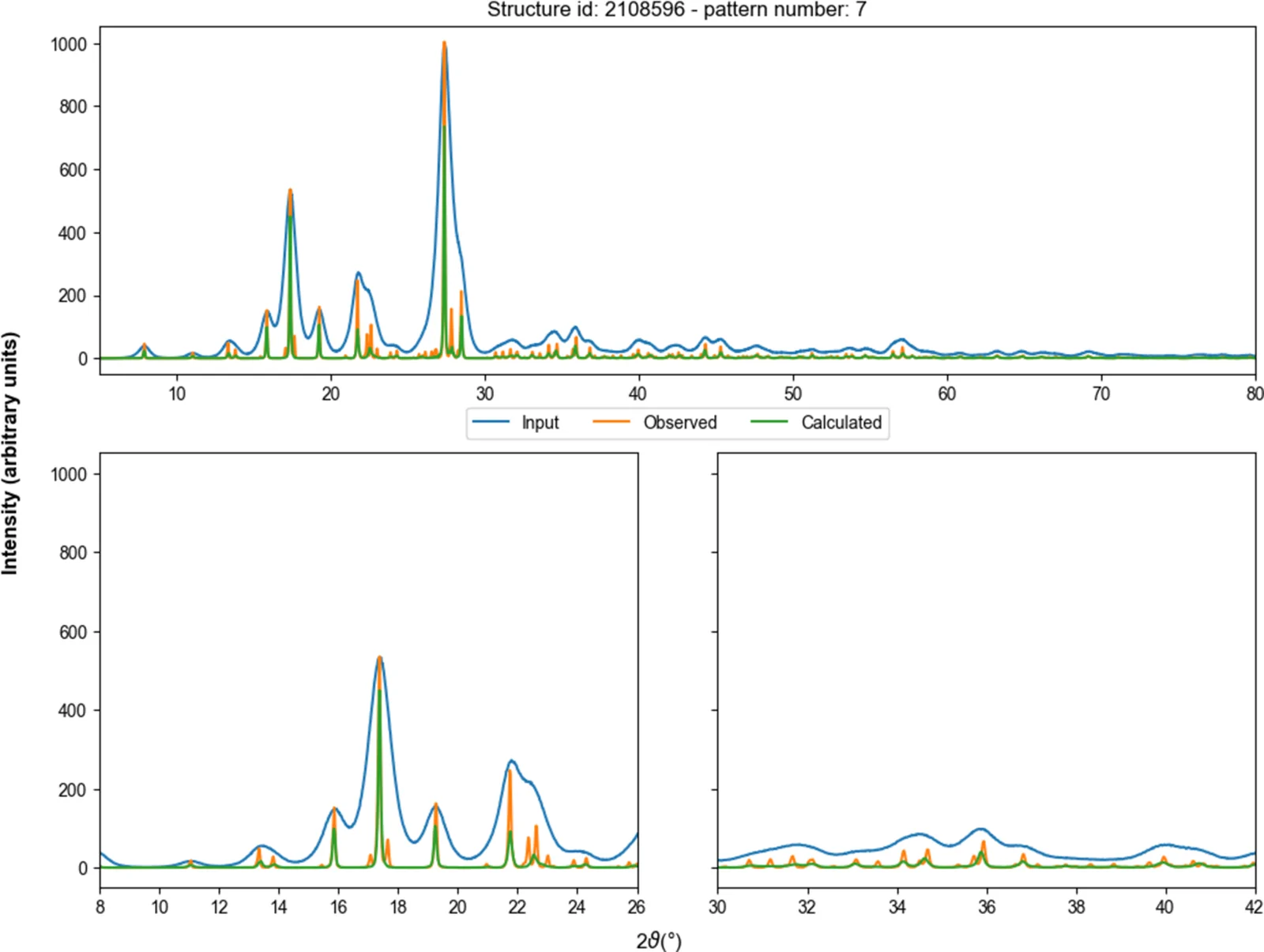
Overlay of input (in blue), observed (target, in orange) and calculated (predicted, in green) spectra. The top panel presents the full-range profiles. The bottom panels focus on two magnified spectral regions.

To provide a representative visual example, the profile whose prediction was closest to the average performance across the test set was selected. This corresponds to the structure with COD ID 2108596 and pattern number 7, as shown in Fig. 3 ▸.

The test set comprises 10% of the original dataset and is completely separate from both the training and validation sets. For each test structure, *POWnet* generated nine output predicted diffraction profiles numbered 2 to 10 corresponding to the input simulated patterns. This resulted in nine peak-width classes, PW_*i*_ (with *i* from 2 to 10), each containing 3313 patterns. The *POWnet* results for each class were compared with those obtained from the corresponding class of input simulated patterns, which were provided to the network. The input simulated pattern numbered 1 served as the reference profile for *POWnet*.

Both the simulated patterns and those generated by *POWnet* were subjected to the structure-solution process using the *EXPO* software in its default mode, and performance was evaluated according to the criteria described in the previous section. Before analyzing the results, it is important to emphasize that the determination of unit-cell parameters is the preliminary and fundamental step of the solution process. If the unit cell is incorrect or unidentified, crystal structure determination becomes impossible. This explains why cell determination is crucial; many crystal structures remain unsolved simply because their unit cell could not be identified, especially when data quality is poor.

A key parameter reflecting the quality of XRPD data, specifically peak resolution, is the mean FWHM (°) of the peaks in the pattern. For each PW_*i*_ class (with varying *i*), the mean FWHM values were calculated for both the input profiles and the corresponding *POWnet* output profiles within that class. The FWHM values were obtained using an automatic profile-fitting procedure implemented in the peak search tool available in *EXPO*.

**Figure 4:**
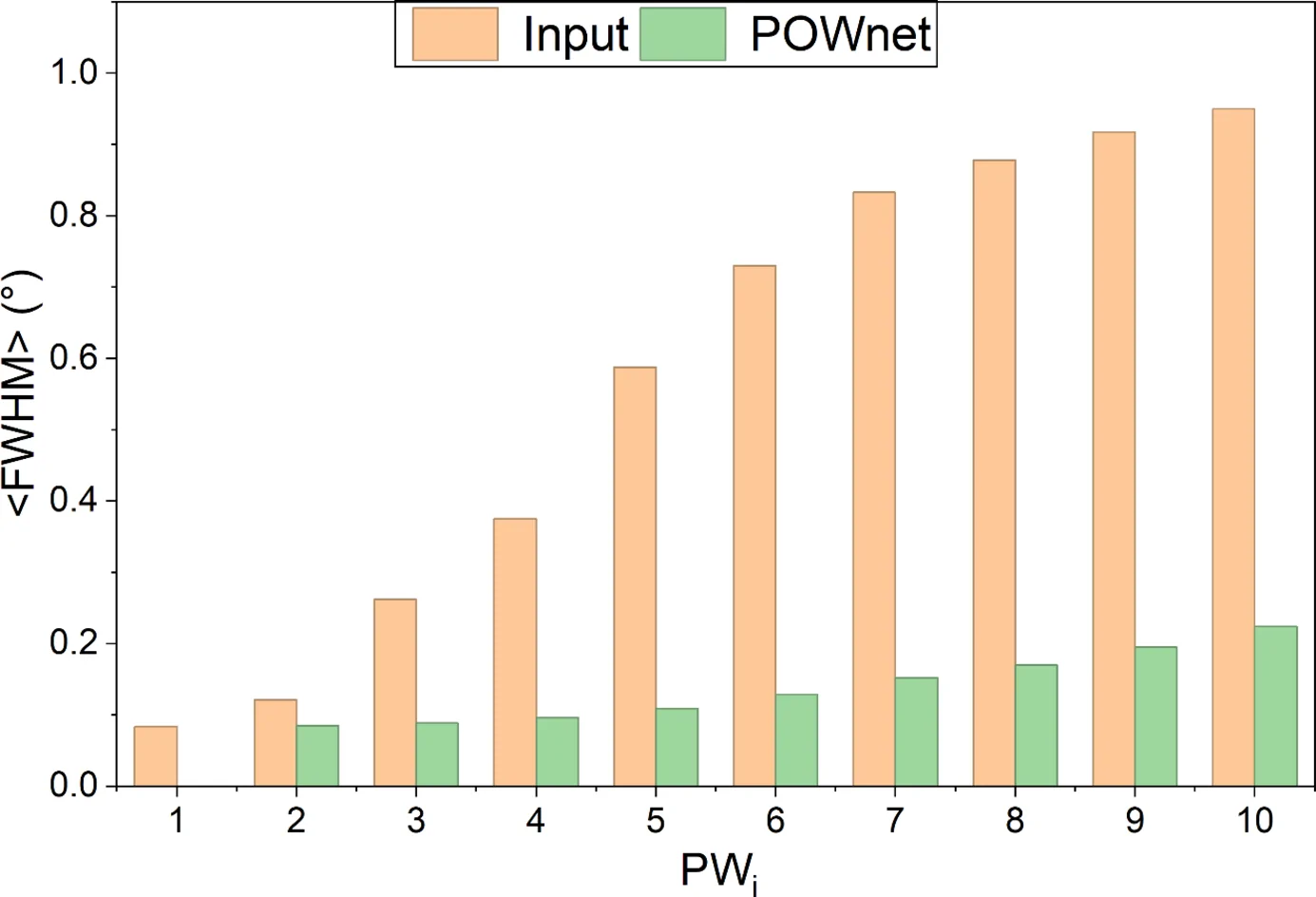
The mean FWHM (°) calculated for input and *POWnet*-generated profiles for each PW_*i*_ class (with *i* from 1 to 10).

These mean FWHM values are reported in Fig. 4 ▸. The average FWHM value corresponding to the PW_1_ class serves as the ideal reference, representing conditions most favorable for successful structure solution.

The results show that 〈FWHM〉 remains approximately constant (in the range 0.08°–0.22°) across the different classes of *POWnet* profiles, while it increases with increasing the peak width in the classes of input profiles. This demonstrates that the patterns produced by *POWnet* maintain stable and high-quality peak widths regardless of class, and are therefore better-suited for the structure-solution process.

#### 3.1.1. Indexing

**Figure 5:**
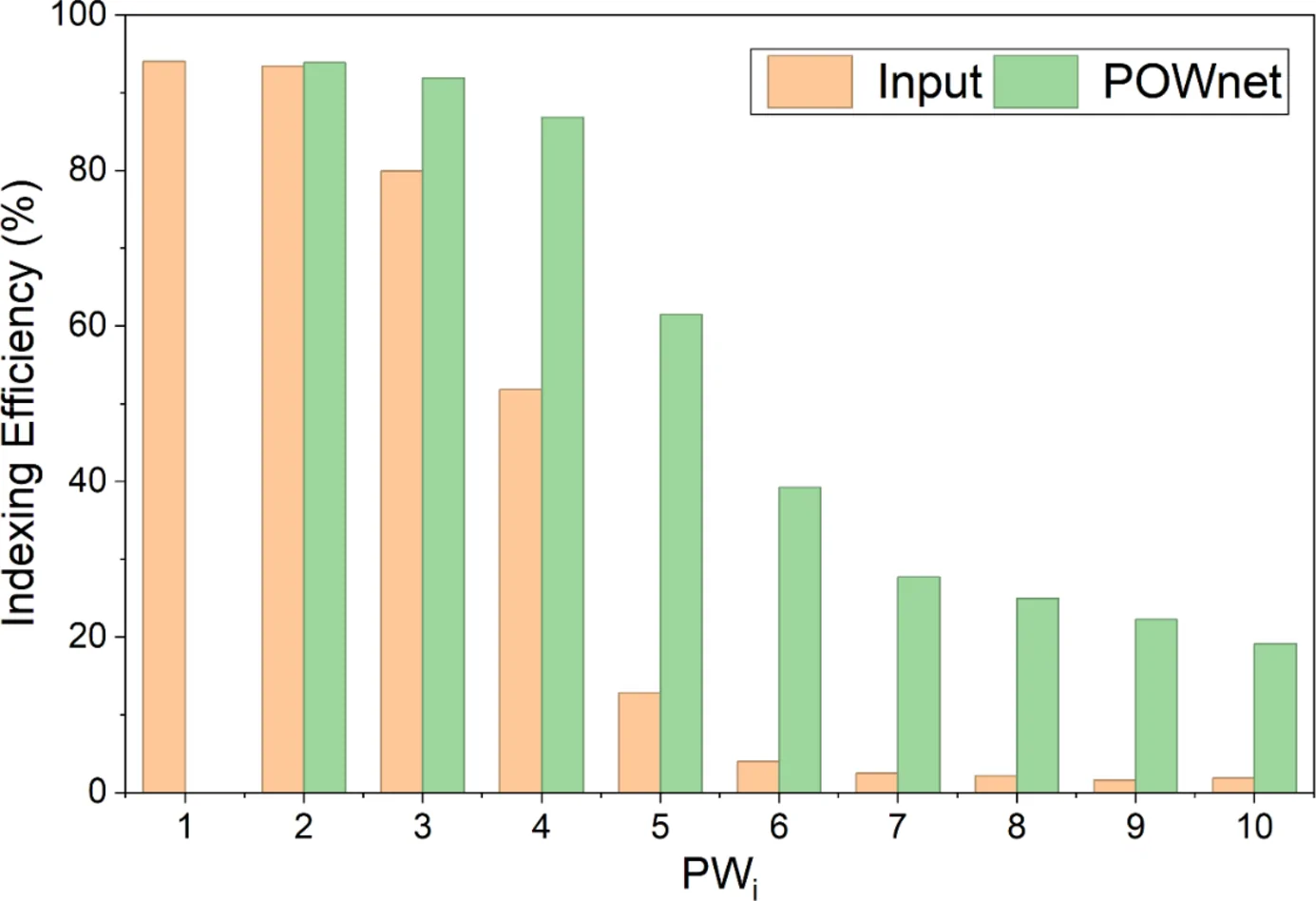
Indexing efficiency values for input and *POWnet* output profiles as a function of the PW class.

**Figure 6:**
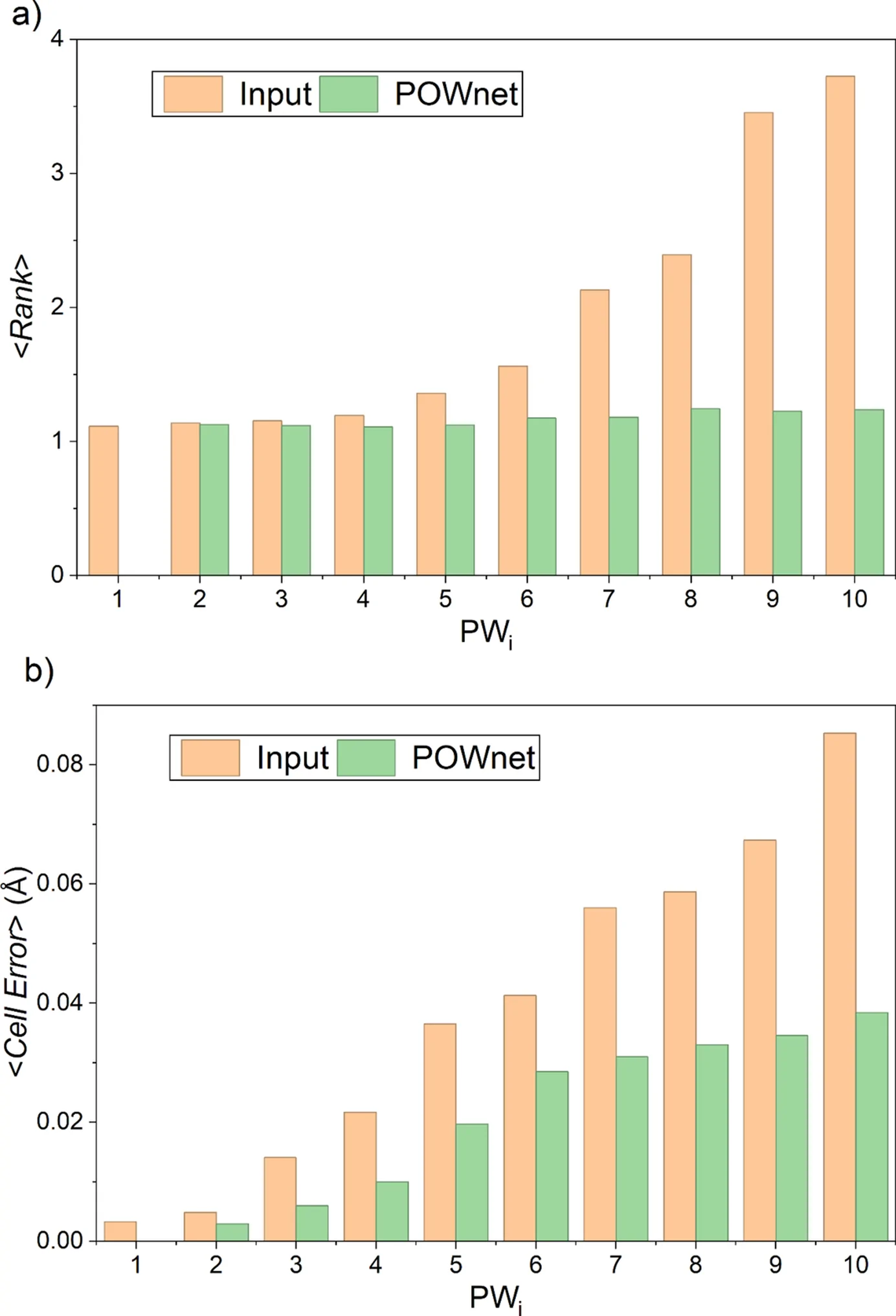
The mean (*a*) rank and (*b*) cell error (Å) for input and *POWnet* profiles as a function of the PW class.

We evaluated three main indexing parameters for the input and output profiles. The first is the indexing efficiency, defined as the percentage of cases in which a unit cell similar to the published one appears in the list of candidate cells obtained by *EXPO*. The indexing-efficiency values for input and *POWnet* output profiles, across the different PW classes, are shown in Fig. 5 ▸. The second parameter is the mean rank, corresponding to the position of the correct identified unit cell within the candidate list, averaged over all profiles in the class. The closer this value is to one, the more reliable the indexing process. The mean rank values are shown in Fig. 6 ▸(*a*). The third parameter is the cell error (Å), defined as the MSE between the lengths of the three unit-cell axes correctly identified (when present in the candidate list) and those of the published unit cell. This metric was averaged across all patterns in the class and is presented in Fig. 6 ▸(*b*) for input and *POWnet*-predicted profiles as a function of PW class. An average cell error close to 0.0 Å indicates the highest accuracy.

All three indexing parameters indicate that, for the input profiles, the values change significantly and the quality of cell determination deteriorates markedly as the peak width increases. For the *POWnet* profiles, the parameters show similar trends, although the variations are less pronounced, with efficiency values at the largest peak width (PW_10_) remaining close to 20% (whereas the corresponding value for input profiles is less than 2%). This is a remarkable finding: *POWnet* is able to generate powder patterns suitable for cell-parameter determination by standard indexing methods, with a non-negligible probability, even when starting from the typically low-quality data obtained from nanocrystals.

We observe that, for the correctly identified unit cells, the average rank obtained from the simulated profiles increases with increasing PW_*i*_, whereas it remains systematically equal to 1 for the profiles predicted by *POWnet*, as for the ideal generated profiles (PW_1_). To support this observation, we calculated the average number of diffraction peaks detected by the automatic peak-search procedure for simulated patterns corresponding to PW_1_ (48), for all other PW_*i*_ classes (*i* = 2–10) (27) simulated profiles, and for all *POWnet*-generated profiles (35). The superiority of the indexing process applied to the *POWnet*-predicted patterns is also confirmed by the larger number of well resolved diffraction peaks, which leads to a rank consistently equal to 1 and indicates that the indexing figure of merit is more significant.

**Figure 7:**
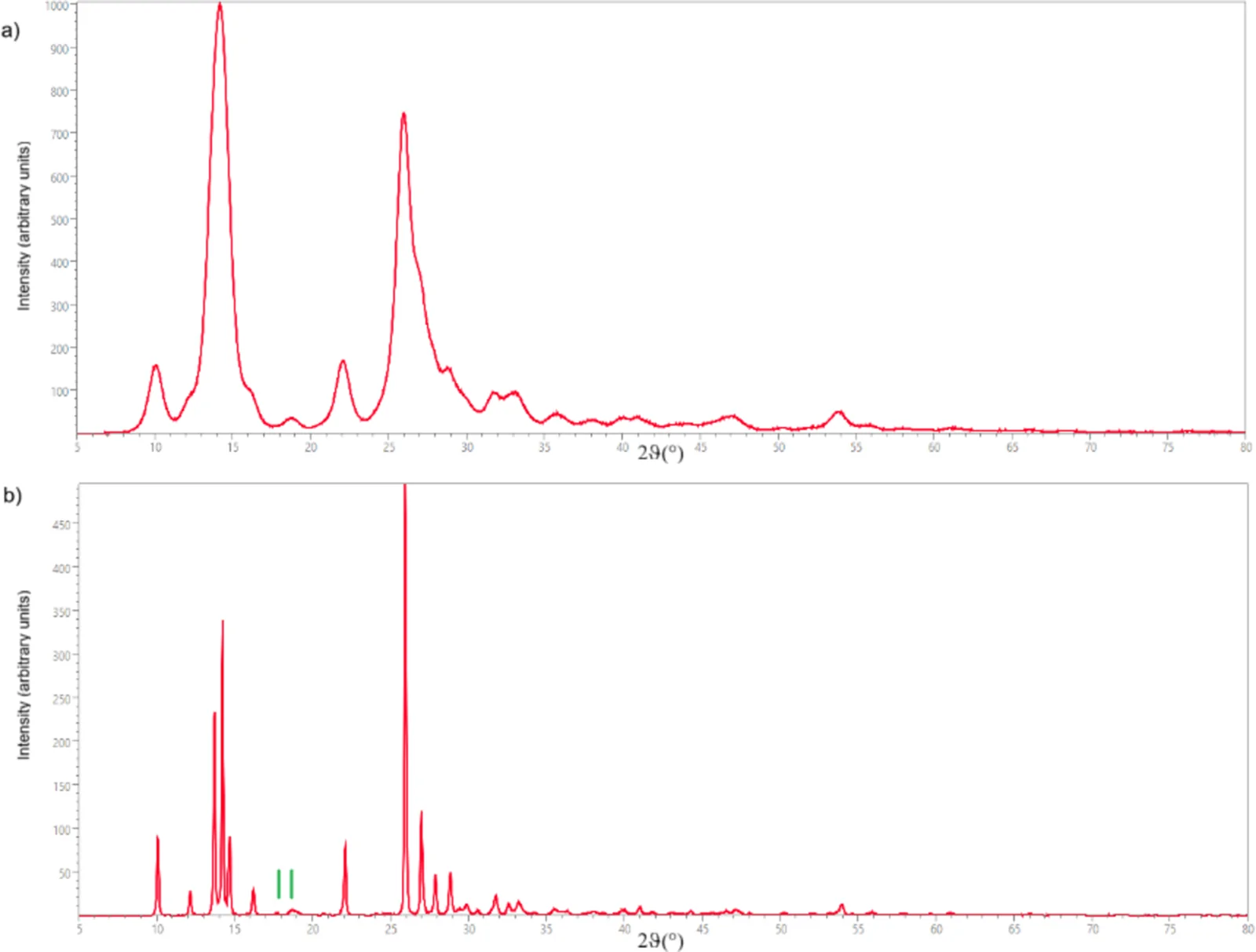
Example of (*a*) a simulated diffraction profile with a very large average FWHM, for which indexing is unsuccessful using both standard and non-standard procedures, together with (*b*) the corresponding predicted profile, which is successfully indexed using non-standard options but not with the default settings.

The indexing process and the results reported in Figs. 5 ▸ and 6 ▸ were obtained through default runs of *EXPO* on both simulated patterns provided to *POWnet* and predicted patterns generated by *POWnet*. These runs included the default peak-search step, which is performed prior to cell search. Examining the details, we observe that while the indexing of simulated patterns generally does not also improve when non-default options are applied, performance on predicted patterns can improve under non-standard runs. In Fig. 7 ▸, we show an example of an input simulated profile with a very large average FWHM value (FWHM ≃ 1 Å) [Fig. 7 ▸(*a*)] and its corresponding *POWnet*-predicted profile [Fig. 7 ▸(*b*)]. While the indexing process fails under many non-default peak-search and cell-search attempts with the input profile, the correct cell is obtained simply by modifying the peak-search threshold when using the predicted pattern. This adjustment allows the low 2ϑ-angle peaks highlighted in green in Fig. 7 ▸(*b*) to be excluded in the indexing process, ultimately leading to the correct cell determination, as the selection of accurate low 2ϑ-angle peak positions is crucial for successful indexing.

#### 3.1.2. Extraction

Another important step in the *ab initio* structure-solution process is the extraction of integrated reflection intensities from the powder diffraction pattern. This step is crucial because the integrated intensities, and thus the experimental structure-factor moduli, constitute the key information used in the phasing process and in the calculation of the electron-density map from which the atomic positions are determined.

The greater the peak overlap, the less accurate the extracted intensities, and consequently, the lower the probability of successfully solving the structure.

An indicator of the quality of a diffraction pattern, and of its suitability for structure solution, is the *R*_F_ factor described in Section 2.5, which measures the average discrepancy between the extracted structure-factor moduli and those calculated from the corresponding published structural model reported in the CIF. A low *R*_F_ value indicates reliable extraction and intensities suitable for successful phasing and structure determination. However, peak overlap, whether minor or significant, inevitably introduces errors into the experimentally extracted intensities, causing *R*_F_ to increase as peak overlap becomes more severe.

**Figure 8:**
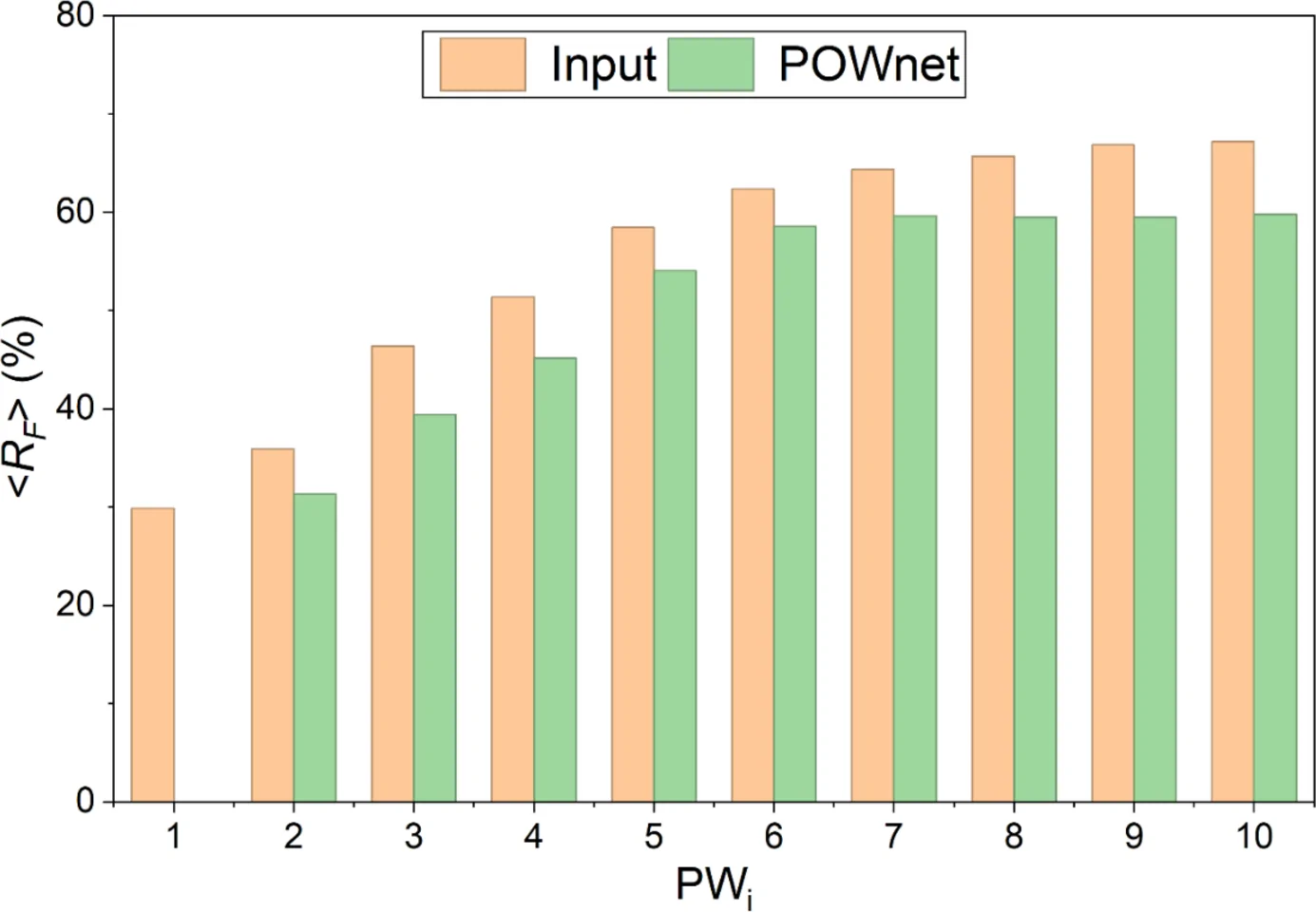
〈*R*_F_〉 (%) for input and *POWnet* profiles within each PW_*i*_ class (with *i* from 1 to 10).

We calculated *R*_F_ (%) for each simulated and predicted profile, as well as the corresponding average values within each peak-width class. These average values are presented in Fig. 8 ▸. The results indicate that, although 〈*R*_F_〉 values increase with increasing peak width, the values obtained for the output profiles remain lower than those of the corresponding input profiles, even at large peak widths. This clearly demonstrates that the integrated diffraction intensities extracted from the predicted profiles are less affected by errors than those obtained from the corresponding simulated patterns, even when substantial peak broadening is present. Notably, the best 〈*R*_F_〉 value for *POWnet* profiles with narrow peaks (PW_1_ class) is not close to zero but ∼30%, indicating that peak overlap, even when not severe, systematically compromises the extraction step. When peak overlap is severe, while the worst 〈*R*_F_〉 value for the input profiles approaches 67% at the maximum peak width, the corresponding value for the output profiles remains below 50%.

#### 3.1.3. Structure solution

A typical *ab initio* structure-solution process concludes with the identification of atomic positions within the asymmetric unit of the crystal cell. These positions are obtained through a peak-search procedure applied to the electron-density map, which is calculated using the experimental structure-factor moduli extracted from the experimental pattern and associated with each reflection, together with the corresponding phases estimated probabilistically by direct methods.

The success of the phasing process strongly depends on the accuracy of the extracted integrated intensities, while the quality of the resulting electron-density map is influenced by both the reflection intensities and the estimated phases. For a structure that is already known, a practical way to assess the reliability of the solution process is to evaluate the ratio between the number of correctly located atomic positions in the asymmetric unit, typically defined as those lying within 0.6 Å of the reference positions, and the number of published atomic positions reported in the corresponding CIF (CLP parameter).

**Figure 9:**
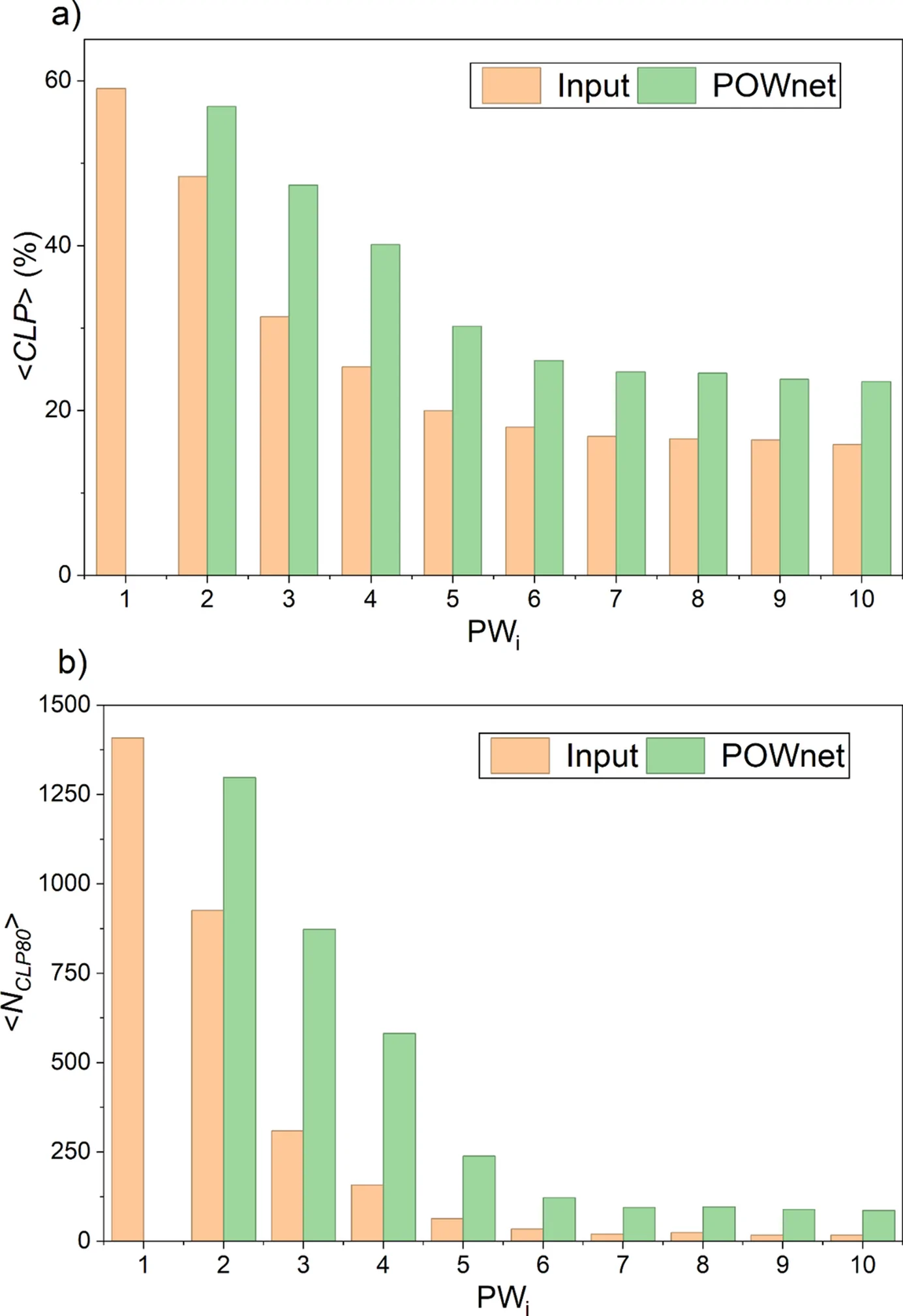
(*a*) Average ratio between correctly located atoms in the asymmetric unit and the number of published atomic positions reported in the CIF for each test structure (〈CLP〉), and (*b*) number of structure solutions for which more than 80% of the atoms have been correctly located by the automatic structure-solution process using both input and *POWnet* profiles (*N*_CLP80_), for each peak-width class, calculated from the input and *POWnet*-predicted profiles. A total of 1574 correct solutions for the input profiles (summed over the PW_*i*_ classes, *i* = 2–10), corresponding to 970 solved structures out of 3313, and 3483 for the output profiles, corresponding to 1482 solved structures.

In this study, *ab initio* structure solution in reciprocal space by direct methods was performed automatically using *EXPO* on both simulated diffraction patterns and predicted ones. The resulting average CLP values for input and *POWnet*-generated profiles within each PW_*i*_ class (with *i* from 1 to 10) are reported in Fig. 9 ▸(*a*). Subsequently, the number of solutions with CLP values greater than 80%, *N*_CLP80_, corresponding to approximately complete structure solutions, was calculated for both input and output profiles. The analysis yielded 1574 correct solutions for the input profiles (summed over the PW_*i*_ classes, *i* = 2–10), corresponding to 970 solved structures out of 3313. For the output profiles, the number of correct solutions increased to 3483, corresponding to 1482 solved structures. These results are shown by class in Fig. 9 ▸(*b*) and can be compared with the 1409 correct solutions obtained for the PW_1_ class. This represents a substantial improvement when moving from profile generation to profile prediction, both in the number of profiles leading to a correct solution and in the total number of successfully solved structures.

In general, the probability of achieving a successful fully automatic *ab initio* structure solution from powder diffraction data using direct methods is relatively low, and manual or semi-automatic intervention is often required to obtain the correct structure. This challenge is particularly pronounced for organic compounds, which exhibit low scattering power, irrespective of whether the number of atoms in the asymmetric unit is small (≤15) or large. In our database of 33 125 structures, the number of non-hydrogen atoms (since hydrogen atoms contribute negligibly to X-ray diffraction) in the asymmetric unit ranges from 5 to 35.

Consequently, even the 〈CLP〉 value associated with the smallest peak width (PW_1_), which ideally represents the most favorable case with minimal peak overlap, remains well below 100%. Factors such as peak overlap, structural complexity, diffraction-pattern quality and crystal symmetry all contribute to the intrinsic difficulty of the *ab initio* solution process. Structure solution in direct space, for example by simulated annealing in *EXPO*, is often a valid alternative to direct methods, especially for organic compounds. However, since simulated annealing requires additional structural information, such as the expected molecular geometry, it is less suitable for large-scale automated runs. For this reason, all results presented in this work were obtained using the default automated direct methods implementation in *EXPO*. Nevertheless, the results can be significantly improved by applying non-standard structure-related options.

As expected, for the input profiles, 〈CLP〉 values decrease markedly with increasing peak width. In contrast, for the *POWnet* profiles, although 〈CLP〉 also declines as the peak width increases, the reduction is less severe. Remarkably, even under the most unfavorable condition (PW_10_), 〈CLP〉 is ≃23% for *POWnet* predictions, indicating a greater probability of successfully solving a crystal structure even when starting from a diffraction profile comparable to that shown in Fig. 7 ▸(*a*). In addition, the *N*_CLP80_ values for the *POWnet* profiles are higher than those of the corresponding input profiles across all peak-width classes. This represents an unprecedented and promising result for the characterization of polycrystalline and nanocrystalline materials, as it demonstrates, for the first time, that *ab initio* crystal structure solution is not absolutely precluded in these challenging cases.

### 3.2. Experimental profiles

Although experimental patterns we retrieved from COD did not exhibit severe peak broadening and peak asymmetry, this test was designed to further confirm that *POWnet*-predicted profiles are generally better suited for default *ab initio* structure determination and that *POWnet* constitutes a valuable tool for tackling structure-solution challenges. The peaks of predicted profiles have FWHM values systematically smaller than their corresponding experimental patterns (see Table S1). Both experimental and corresponding *POWnet* output patterns were subjected to automatic indexing, extraction and structure-solution procedures in order to assess their suitability for structure determination. Table 3 ▸ summarizes the parameters introduced in the previous sections for the indexing, structure-factor modulus extraction and structure-solution steps, for both experimental and *POWnet* profiles.

The performance of the structure-solution steps obtained using the predicted profiles improves, indicating that *POWnet* is capable of generating diffraction patterns that increase the probability of successfully solving powder structures. All calculations were carried out fully automatically. We therefore expect that the observed improvement could be further enhanced through the adoption of *ad hoc* strategies during both the indexing and structure-solution steps.

**Figure 10:**
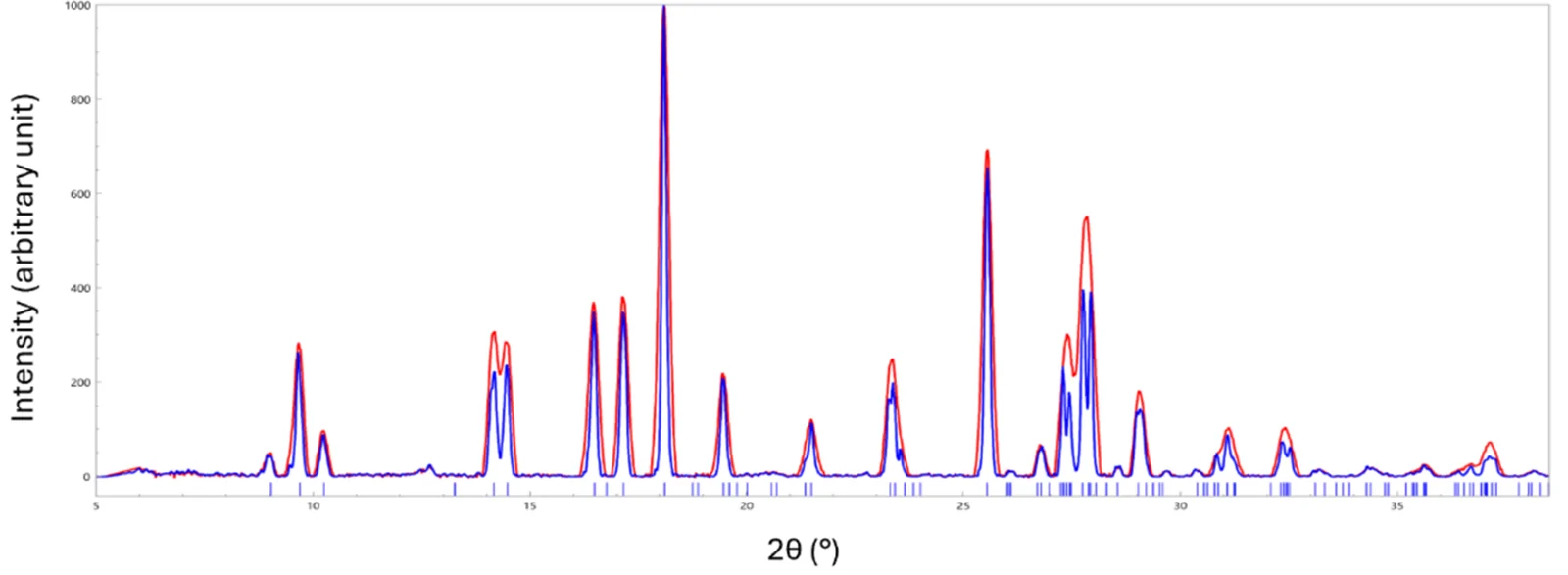
Experimental (red line) and *POWnet* (blue line) profiles for the structure with COD ID 7122535 in a selected range. Reflection positions are shown at the bottom by small blue vertical lines.

In Fig. 10 ▸, for the structure with COD ID 7122535, we present a detailed view of a selected range of the experimental (red line) and *POWnet*-predicted (blue line) profiles, with the corresponding reflection positions shown at the bottom by small blue vertical lines. This comparison highlights the ability of *POWnet* to improve peak resolution even under average-quality conditions typical of standard laboratory data and for a well crystallized structure. Such improvements are overall beneficial for the structure-solution process.

### 3.3. Statistical analysis of deep-learning results

Results obtained by the structure-solution process applied to *POWnet* profiles were used to build a regression model for a double purpose: (i) identifying the main variables that influence the outcome of the structure solution and (ii) optimizing the application of the *POWnet* integrated approach, by predicting possible outcomes of the *POWnet* pre-processing + structure-solution pipeline. To these aims, results from simulated profiles were used as a calibration set to train the regression model and to identify key variables for the model predictions, while results from experimental profiles were used as a test set to make predictions based on structural features that are readily accessible after synthesis and X-ray measurement.

The variables chosen to capture the main features of the input data and corresponding crystal structures (*X* variables) and those to assess the performance of the *POWnet* + structure-solution pipeline (*Y* variables) are listed in Table 4 ▸. The first *Y* variable (*R*_F_) evaluates the extraction process (see Section 3.1.2), while the second (CLP) is related to the extent of the atomic fragment correctly located at the end of the structure-solution process (see Section 3.1.3).

**Figure 11:**
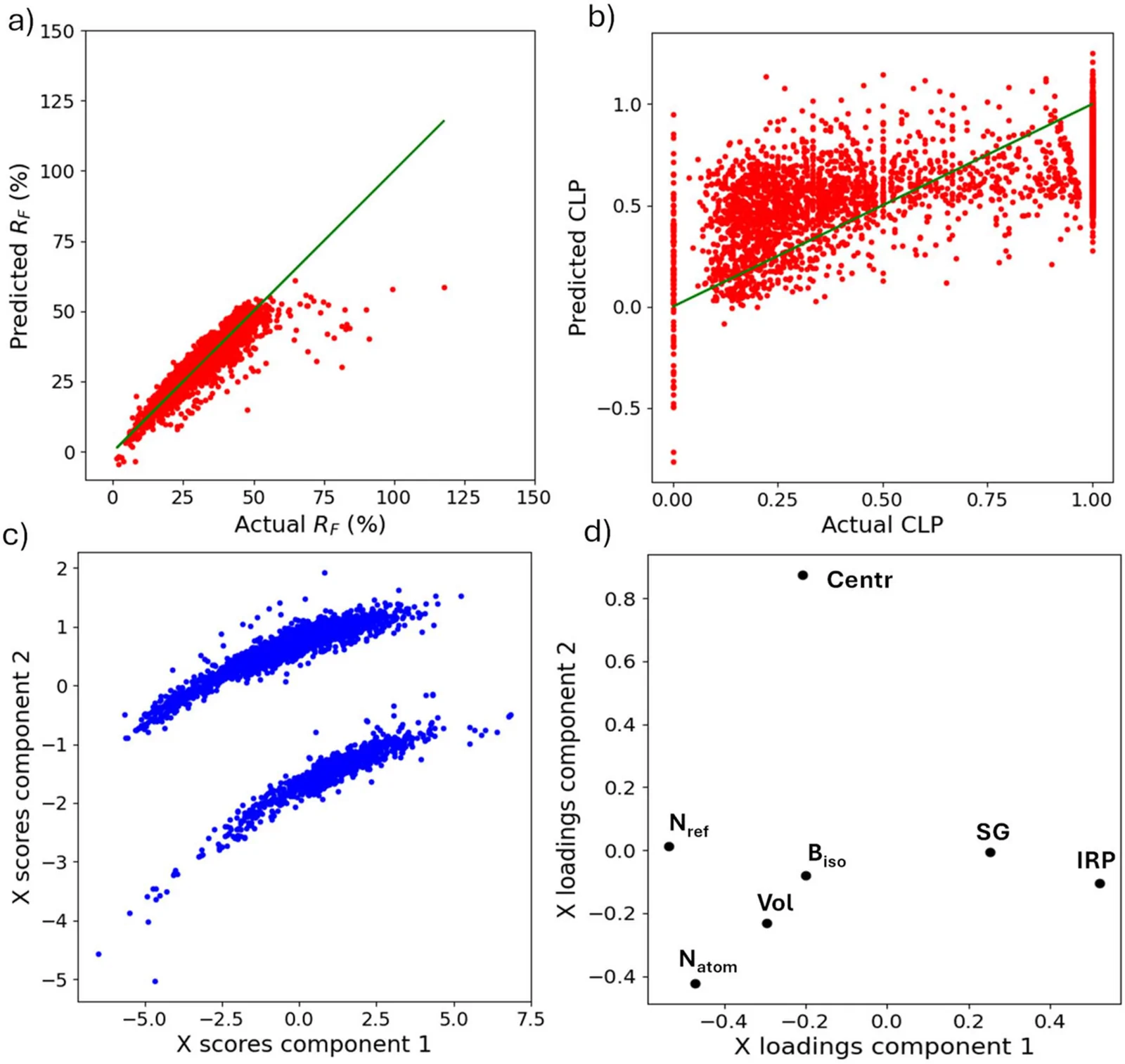
Results of the regression model build on 3313 test simulated XRPD profiles processed by *POWnet*. Scatter plots of the predicted versus actual values of the (*a*) *R*_F_ and (*b*) CLP *Y* variables. The bis­ector green line is shown to guide the eye. (*c*) Scores and (*d*) loadings plots of the *X* variables in the first two principal components PC1 and PC2, which explain 52% and 17%, respectively, of the total data variance. The acronyms of the *X* variables in (*d*) are the same as those reported in Table 4 ▸.

The calibration set for the regression model was formed by the 3313 simulated profiles, where the *X* variables refer to the corresponding crystal structures, while the *Y* variables were extracted from the output of the *POWnet* pre-processing + structure-solution procedure applied to profiles belonging to the peak-width class PW_2_. The quality of the regression model can be inspected by considering Fig. 11 ▸, where predicted versus actual *Y* values are plotted separately for *R*_F_ [Fig. 11 ▸(*a*)] and CLP [Fig. 11 ▸(*b*)]. The predicted *R*_F_ are strongly reliable up to 60%, while the model fails for higher values. A good correlation between predicted and observed values is also obtained for CLP values, with a clear separation between fully recovered structures (CLP = 1) and fully missing structures (CLP = 0). The overall performance of the regression model has an explained variance *R*^2^ = 0.60 and an MSE = 11.3.

An analysis of the scores and loadings of the *X* variables for the first two principal components, which globally explains 69% of the total data variance, is informative for understanding the role of each variable in the regression model. For example, for the first principal component (PC1), the key variables are those located at the extremes of the loading plot along the *x* axis [Fig. 11 ▸(*d*)], that is, the percentage of independent reflections (IRP) for positive loadings and the number of reflections (*N*_ref_) and atoms (*N*_atom_) in the asymmetric units of the reciprocal and direct space, respectively, for negative loadings. This indicates that the key factor in explaining the outcome of the *POWnet* integrated approach is related to the size of the crystal structure and, consequently, to the degree of peak overlap in the XRPD profile. These variables drive the separation of data points along PC1 in the scores plot of the *X* variables [Fig. 11 ▸(*c*)]. The second principal component (PC2) is instead dominated by the centric/acentric character of the space group (Centr). In fact, the score plot in Fig. 11 ▸(*c*) shows two clearly distinct clusters corresponding to structures that possess or lack a center of symmetry.

**Figure 12:**
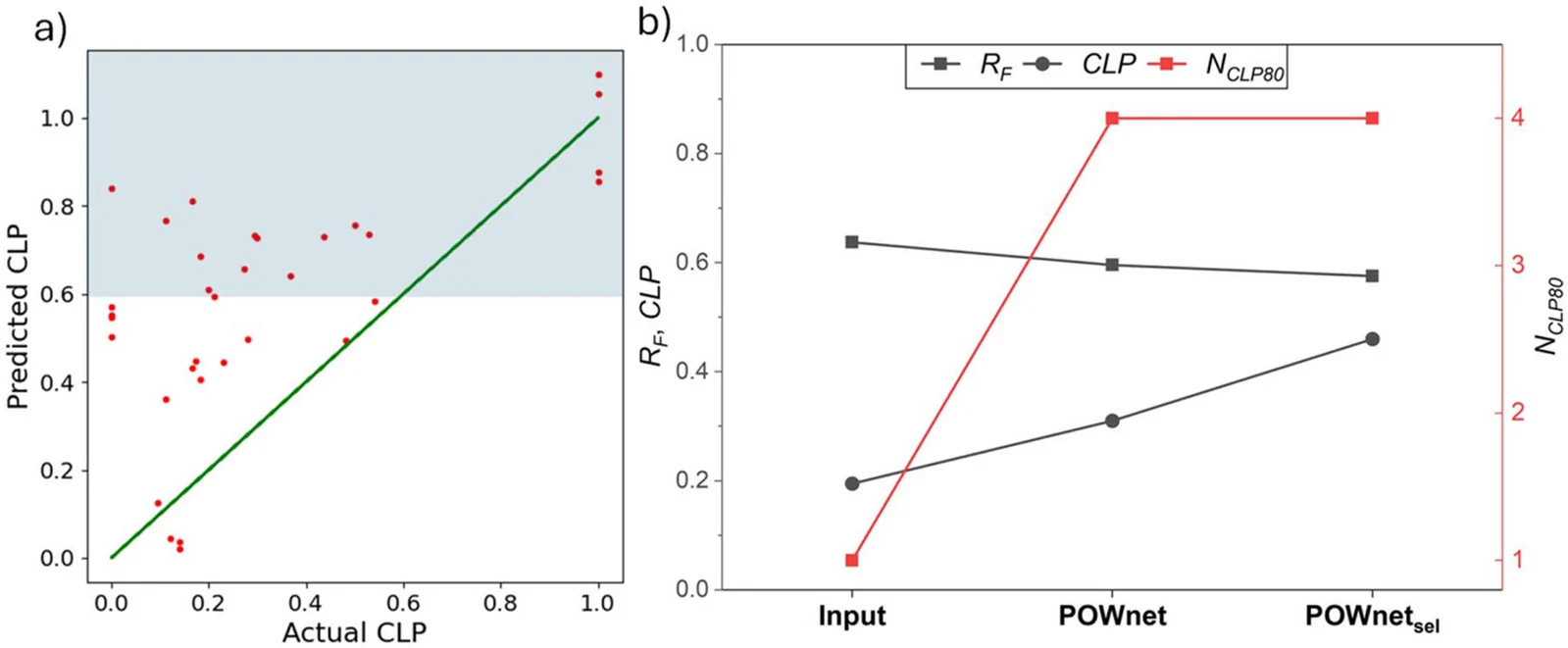
Results of the regression model applied to the 33 experimental XRPD profiles processed by *POWnet*. (*a*) Scatter plot of the predicted versus actual values of the CLP variable. The bis­ector green line is shown to guide the eye. (*b*) Comparison of *R*_F_, CLP and *N*_CLP80_ values obtained for the 33 input and *POWnet* profiles with the 16 profiles selected by the regression model (predicted *R*_F_ > 0.60).

The regression model was then applied to the 33 experimental profiles to predict the outcome of the *POWnet* + structure-solution pipeline, based on the main structural features of the corresponding crystal structures. The CLP model predictions are shown in Fig. 12 ▸(*a*) against the CLP values actually obtained by applying the pipeline. Given the rough correlation shown in Fig. 12 ▸(*a*), the application of the *POWnet* integrated approach can be optimized by introducing a decision-making step based on structural features. A threshold value of 0.6 was then applied to predicted CLP values to select the most promising profiles, and in fact, with this criterion, 16 experimental profiles were identified, whose *R*_F_, CLP and *N*_CLP80_ average values are better than those of the original set [Fig. 12 ▸(*b*)].

## 4. Conclusions

We propose a deep-learning model capable of transforming diffraction patterns from crystalline powder samples, particularly those with small crystallite sizes, enhancing their structural information content and effectively approximating the condition of an ideal perfectly crystalline material measured without instrumental peak-broadening effects. The model is trained across different crystallite sizes, making it applicable to a wide range of real cases. The proposed pipeline, in which a deep-learning model prepares XRPD profiles for optimal execution of the crystal structure determination workflow implemented in the crystallographic software *EXPO*, proved highly effective. It enables the solution of crystal structures from XRPD profiles that are intractable using conventional crystallographic methods. In particular, the proposed approach extends the limits of applicability of *ab initio* solution methods for powder diffraction.

The advantage over other deep-learning-based phasing methods (Larsen *et al.*, 2024 ▸) is that our model does not depend on crystal symmetry or unit-cell parameters, and can be applied to any powder sample, including samples with small crystallite size or those affected by structural imperfections. At the same time, it can be specifically trained to handle specific classes of materials, such as zeolites, MOFs, organics, and so forth.

Of course, this tool must be used with caution as it provides a structural model representing the sample under idealized conditions rather than reflecting its real state. Disorder, crystal imperfections and other effects should be reintroduced *a posteriori* by refining the structural model against the original powder diffraction profile.

Nevertheless, we believe that the proposed approach has the potential to revolutionize the structural characterization of materials by powder diffraction, in both static (*ex situ*) and dynamic (*in situ*) conditions. It opens the way to *ab initio* determination of structural details at atomic resolution of nanomaterials and to investigation of their evolution under external stimuli (Palin *et al.*, 2015 ▸).

Echoing Neil Armstrong’s famous words, we can say: ‘That’s one small step for Crystallography, one giant leap for structural material science.’

With this in mind, we believe that the approaches and ideas presented in this paper can be suitably adapted to address other issues affecting XRPD data, such as limited experimental resolution, as expressed by the minimum interplanar distance. Since atomic-level resolution is a fundamental requirement for successful *ab initio* structure solution, our next focus will be on this challenge, aiming to improve experimental data resolution with the valuable support of deep learning.

## Supplementary Material

Table S1. DOI: 10.1107/S2052252526008444/zx5037sup1.pdf

## Figures and Tables

**Table 1:** Ranges of the FWHM profile parameters *W*, *V* and *U* used to generate the ten diffraction profiles (Profiles 1–10) from each CIF

Number of profile	*W* range		*V* range		*U* range	
1	0.005	0.01	−0.001	0.002	0.005	0.01
2	0.01	0.02	−0.002	0.004	0.010	0.02
3	0.05	0.1	−0.003	0.006	0.015	0.03
4	0.1	0.2	−0.004	0.008	0.020	0.04
5	0.3	0.4	−0.005	0.010	0.025	0.05
6	0.5	0.6	−0.006	0.012	0.030	0.06
7	0.7	0.8	−0.007	0.014	0.035	0.07
8	0.8	0.9	−0.008	0.016	0.040	0.08
9	0.9	1.0	−0.009	0.018	0.045	0.09
10	1.0	1.1	−0.010	0.020	0.050	0.10

**Table 2:** Performance metrics based on the figures of merit across profile resolutions The values represent the mean ± standard deviation computed over the test set, including 3313 samples. The last row shows the performance values independent of the FWHM of the profiles and averaged over 29 817 predictions.

FWHM	*R*_p_	*R*_wp_	Corr	Sim
2	0.111 ± 0.045	0.148 ± 0.065	0.996 ± 0.003	0.998 ± 0.001
3	0.180 ± 0.063	0.221 ± 0.077	0.991 ± 0.010	0.996 ± 0.005
4	0.253 ± 0.088	0.303 ± 0.110	0.979 ± 0.024	0.989 ± 0.014
5	0.402 ± 0.118	0.470 ± 0.144	0.935 ± 0.061	0.959 ± 0.041
6	0.512 ± 0.134	0.585 ± 0.164	0.888 ± 0.092	0.924 ± 0.067
7	0.569 ± 0.131	0.637 ± 0.158	0.857 ± 0.104	0.900 ± 0.078
8	0.588 ± 0.130	0.648 ± 0.148	0.852 ± 0.101	0.897 ± 0.076
9	0.618 ± 0.131	0.668 ± 0.141	0.844 ± 0.101	0.890 ± 0.077
10	0.661 ± 0.129	0.704 ± 0.141	0.823 ± 0.110	0.873 ± 0.086

Avg	0.433 ± 0.224	0.487 ± 0.239	0.907 ± 0.102	0.936 ± 0.075

**Table 3:** Indexing, structure-factor modulus extraction, and structure-solution parameters for 33 experimental and corresponding *POWnet* output profiles

	Experimental	*POWnet*
Indexing		
Indexing efficiency (%)	69.7	81.8
〈Rank〉	1.26	1.11
〈Cell error〉 (Å)	0.017	0.017

Structure-factor modulus extraction		
〈*R*_F_〉 (%)	63.8	59.6

Structure solution		
〈CLP〉 (%)	19.5	31.0
*N*_CLP80_	1	4

**Table 4:** Variables used to characterize the crystal structure (*X* variables) and to assess the performance of the extraction (*R*_F_) and crystal structure solution (CLP) processes

*X* variables	*Y* variables
Unit-cell volume (Vol)	*R*_F_
Space group (SG)	CLP
Number of symmetry-independent reflections (*N*_ref_) (Altomare *et al.*, 1995 ▸)	
Independent reflections percentage (IRP) (Altomare *et al.*, 1995 ▸)	
Centric/acentric (Centr)	
Overall isotropic thermal factor (*B*_iso_)	
Number of atoms in the asymmetric unit (*N*_atom_)	

## Data Availability

The computation data to support the findings of this study are available from https://github.com/f48r1/pownet/tree/main/data. Scripts and experiment results are available at the GitHub link https://github.com/f48r1/pownet.
