# Supplementary material for: New frontiers in the structural analysis of nanomaterials: a deep-learning leap to bridge the gap in crystalline order
Source: IUCrJ. 2026 Oct 8;13(Pt 6):717–32. doi: 10.1107/S2052252526008444 (PMC13763878; doi:10.1107/S2052252526008444)
Supplement: Supplementary file 1 [file m-13-00717-sup1.pdf]

IUCrJ

Volume 13 (2026)

Supporting information for article:

New frontiers in the structural analysis of nanomaterials: a deep-learning leap to bridge the gap in crystalline order

Fabrizio Mastrolorito, Mauro De Feudis, Fulvio Ciriaco, Rocco Caliandro, Orazio Nicolotti and Angela Altomare

Table S1 Main crystal-chemical information for 33 crystal structures retrieved from the Crystallography Open Database (COD), whose experimental diffraction patterns were tested by POWnet. <FWHM$_{exp}$> and <FWHM$_{POWnet}$> denote the mean FWHM values of diffraction peaks calculated from the experimental profile of each test structure and from the corresponding POWnet-generated profile, respectively.

COD ID	Chemical formula	Cell parameters (Å, °)	Space group	<FWHM>$_{exp}$ (°)	<FWHM>$_{POWnet}$ (°)
1502751	C_{18} H_{21} N_3 O_4	8.0467 9.3558 11.9151<br>102.4356 103.1773 98.6481	*P* -1	0.10	0.08
1547319	C_{30} H_{49} N_5 O_6	8.3072 7.8052 25.7246<br>90.0 97.491 90.0	*P* 2_1	0.34	0.14
1550022	C_6 H_6 N_2 O_2	5.2532 5.321 10.9671<br>90.0 101.974 90.0	*P* $2_1/n$	0.13	0.07
1554069	C_6 H_{16} N_2 O_3	5.9349 6.9413 20.5754<br>90.0 90.0 90.0	*P* 2_1 2_1 2_1	0.13	0.08
1554070	C_{12} $H_{29.99}$ N_4 O_5	9.5309 5.2173 17.6011<br>90.0 101.078 90.0	*C* 2	0.50	0.15
1556093	C_9 H_{13} N O_3	13.0075 5.4302 13.94<br>90.0 101.1125 90.0	*P* 2_1	0.26	0.13
1558842	C_{12} H_{16} N_2 O_5	4.9815 30.3259 8.9413<br>90.0 93.776 90.0	*P* $2_1/c$	0.13	0.08
2104632	C_{20} H_{37} N O_3	37.1586 5.3591 5.3097<br>90.0 92.1693 90.0	*P* 2_1	0.08	0.08
2108596	C_{10} H_8 N_4 O_5	11.67 11.4666 8.494<br>90.0 106.848 90.0	*P* $2_1/c$	0.15	0.09
2228277	C_{11} H_8 N_4 O_3	13.8774 14.5321 4.9305<br>90.0 90.83 90.0	*P* $2_1/a$	0.25	0.14
2312003	C_{17} H_{13} N O_4	25.8641 13.2022 4.053<br>90.0 90.0 90.0	*P* 2_1 2_1 2_1	0.12	0.08

2312004	$C_{17} H_{15} N O_5$	25.2195 12.8116 4.5493<br>90.0 90.0 90.0	*P* 2_1 2_1 2_1	0.12	0.09
4129050	$C_{12} H_{11} N O_3$	8.4738 14.0009 11.4724<br>90.0 125.3651 90.0	*P* 2_1/*c*	0.13	0.08
4129065	$C_{10} H_{16} N O$	12.9864 11.7581 11.7294<br>90.0 90.0 90.0	*P b c a*	0.09	0.08
4501722	$C_6 H_{15} N O_2$	6.426 7.1941 10.0442<br>66.6999 84.6295 83.1542	*P* -1	0.12	0.08
4503048	$C_{18} H_8 N_6 O_4$	9.6229 17.813 9.2418<br>90.0 90.0 90.0	*P c a* 2_1	0.13	0.07
4503049	$C_{20} H_{18} N_4 O_8$	11.3988 11.8643 3.8022<br>96.3647 95.9064 108.8978	*P* -1	0.17	0.08
4503050	$C_{18} H_{16} N_6 O_8$	11.2777 12.8578 6.4718<br>90.0 94.697 90.0	*P* 2_1/*c*	0.25	0.11
4503051	$C_{20} H_{10} N_4 O_4$	34.5859 5.7132 8.0155<br>90.0 90.0 90.0	*C m c a*	0.15	0.11
4507474	$C_7 H_{13} N O_2$	13.5616 6.249 9.8058<br>90.0 112.2003 90.0	*P* 2_1/*n*	0.06	0.07
4509268	$C_{15} H_{15} N O_8$	10.5423 10.9487 7.0994<br>107.9097 97.7465 88.0882	*P* -1	0.09	0.08
7105350	$C_9 H_{12} N_4 O_4$	5.2185 8.2901 12.9358<br>95.827 96.854 100.333	*P* -1	0.17	0.09
7107445	$C_6 H_{14} N_4 O_2$	9.7565 16.023 5.5805<br>90.0 98.058 90.0	*P* 2_1	0.24	0.13
7122535	$C_{12} H_{12} N_2 O_6$	10.5364 18.2347 6.9782<br>90.0 111.6043 90.0	*P* 2_1/*c*	0.38	0.14
7123924	$C_{16} H_{18} N_4 O_7$	4.915 11.6639 15.5627<br>95.2473 90.6137 92.843	*P* -1	0.10	0.07
7123925	$C_{16} H_{19} N_5 O_6$	22.9747 10.9249 7.0656	*P* 2_1 2_1 2_1	0.11	0.07

		90.0 90.0 90.0			
7207459	$C_{13} H_{18} N_2 O_4$	9.475 10.1747 14.532<br>90.0 90.0 90.0	$P\,2_1\,2_1\,2_1$	0.17	0.09
7207460	$C_{13} H_{18} N_2 O_4$	9.3224 10.0994 14.716<br>90.0 90.0 90.0	$P\,2_1\,2_1\,2_1$	0.19	0.08
7232573	$C_{30} H_{42} N_6 O_4$	17.05 9.8304 9.3339<br>90.0 105.097 90.0	$P\,2_1/c$	0.12	0.08
7235100	$C_{38} H_{46} N_2 O_{10}$	16.2991 5.8654 18.6876<br>90.0 100.052 90.0	$P\,2_1$	0.07	0.07
7235104	$C_{38} H_{46} N_2 O_{10}$	12.705 5.7631 25.0<br>90.0 95.041 90.0	$P\,2_1$	0.13	0.08
7236273	$C_{11} H_{15} N_2 O_4$	9.0364 18.7601 7.3289<br>90.0 105.84 90.0	$P\,2_1/c$	0.10	0.08
7249693	$C_{31} H_{42} N_8 O_3$	9.6921 15.7727 12.6377<br>90.0 121.623 90.0	$P\,2_1$	0.17	0.08
